# An MM and QM Study of Biomimetic Catalysis of Diels-Alder Reactions Using Cyclodextrins

**DOI:** 10.3390/catal8020051

**Published:** 2018-01-29

**Authors:** Wei Chen, Lipeng Sun, Zhiye Tang, Zulfikhar A. Ali, Bryan M. Wong, Chia-en A. Chang

**Affiliations:** 1 Department of Chemistry, University of California, Riverside, CA 92521, USA; 2 Department of Chemical & Environmental Engineering and Materials Science & Engineering Program, University of California, Riverside, CA 92521, USA; 3 Illinois Rocstar LLC, P.O. Box 3001, Champaign, IL 61826, USA

**Keywords:** flexibility, preorganization, host-guest, nonpolar pocket, design and synthesis, enzyme

## Abstract

We performed a computational investigation of the mechanism by which cyclodextrins (CDs) catalyze Diels-Alder reactions between 9-anthracenemethanol and *N*-cyclohexylmaleimide. Hydrogen bonds (Hbonds) between *N*-cyclohexylmaleimide and the hydroxyl groups of cyclodextrins were suggested to play an important role in this catalytic process. However, our free energy calculations and molecular dynamics simulations showed that these Hbonds are not stable, and quantum mechanical calculations suggested that the reaction is not promoted by these Hbonds. The binding of 9-anthracenemethanol and *N*-cyclohexylmaleimide to cyclodextrins was the key to the catalytic process. Cyclodextrins act as a container to hold the two reactants in the cavity, pre-organize them for the reactions, and thus reduce the entropy penalty to the activation free energy. Dimethyl-β-CD was a better catalyst for this specific reaction than β-CD because of its stronger van der Waals interaction with the pre-organized reactants and its better performance in reducing the activation energy. This computational work sheds light on the mechanism of the catalytic reaction by cyclodextrins and introduces new perspectives of supramolecular catalysis.

## 1. Introduction

Supramolecular catalysts offer potential advantages over enzymes, including greater physical and chemical stability, lower molecular weight, and a far more varied selection of chemical versatility for the creation of structure and functionality. Due to their unique chemical and physical properties, cyclodextrins are useful in pharmaceutical, food, and agricultural industries [1–5]. Their hydrophobic pocket also allows them to catalyze chemical reactions similar to an enzyme [6–12]. In addition, β-cyclodextrins have been used as a catalyst to promote the Diels-Alder reaction and synthesize various compounds [13–16].

The Diels-Alder reaction is an important carbon–carbon bond formation reaction in organic synthesis. It forms two carbon–carbon bonds and up to four new stereo centers in one step. For typical Diels-Alder reactions between a diene and dienophile, frontier molecular orbital theory states that the interaction of the highest occupied molecular orbital (HOMO) of the diene with the lowest unoccupied molecular orbital (LUMO) of the dienophile is the dominant interaction in the transition state [17]. The rate of the Diels-Alder reaction could be accelerated by narrowing the energy gap between the HOMO and LUMO [18,19].

**Figure 1:**
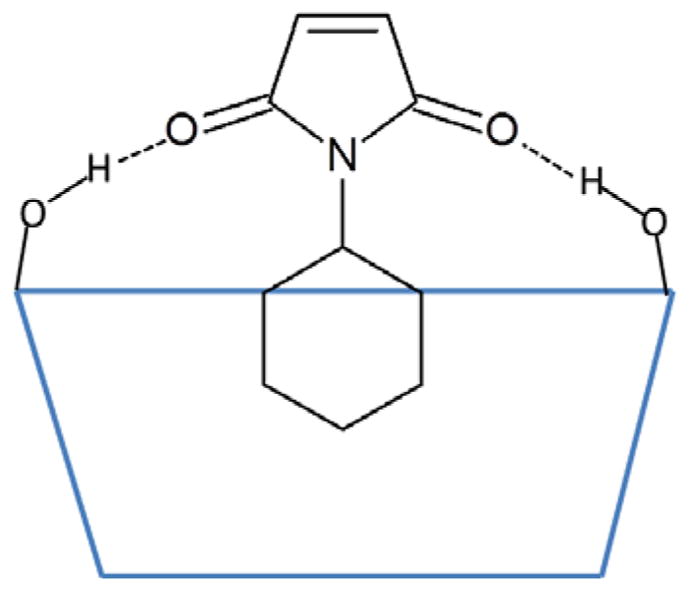
Illustration of the hydrogen bonds between the carbonyl groups on maleimides and the secondary hydroxyl groups on cyclodextrins. *N*-cyclohexylmaleimide is used as an example. The blue trapezoid represents the cyclodextrin, and the dotted lines represent the hydrogen bonds.

Recently, cyclodextrins were reported to promote Diels-Alder reactions of 9-anthracenemethanol with a variety of *N*-substituted maleimides under mild reaction conditions [15]. In this paper, Chaudhuri et al. proposed a mechanism whereby the cyclodextrins bind the hydrophobic substituents on the maleimides (**2a**–**2d**) and activate the dienophile by electronic modulation of the maleimide double bond via hydrogen bonds (Hbonds) with cyclodextrins (Figure 1). Methyl-β-cyclodextrin was found to bind maleimides with large *N*-substituents, such as **2a**, more strongly than β-cyclodextrin, as indicated by the greater changes in the 1H NMR chemical shifts. Methyl-β-cyclodextrin is also significantly more efficient than β-cyclodextrin at promoting the reaction of *N*-cyclohexylmaleimide. The authors further suggested that this situation is likely because methyl-β-cyclodextrin is both more flexible and has a more non-polar cavity than β-cyclodextrin [15]. However, if the reaction is promoted by the Hbonds between the maleimides and cyclodextrins, then β-cyclodextrin should perform at least as well as methyl-β-cyclodextrin since it has more hydroxyl groups to participate in the hydrogen bonding. The mechanism of how the flexibility and hydrophobicity of methyl-β-cyclodextrin aids the catalytic process is also unknown.

**Figure 2:**
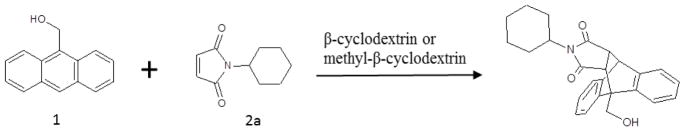
The Diels-Alder reaction of 9-anthracenemethanol (**1**) with N-cyclohexyl maleimide (**2a**), with β-cyclodextrin or methyl-β-cyclodextrin as a catalyst.

A number of studies utilized computational methods to look at the catalytic mechanism of β-cyclodextrin as a catalyst in organic reactions. Luzhov and Venanzi used the Austin Model 1 (AM1) method to study the reaction of phenyl acetate with β-cyclodextrin [20] and found large energy differences between different reaction sites. Eto et al. used the Parameterized Model number 3 (PM3) method to study the pericyclic reaction of cinnamyl xanthates in β-cyclodextrin cavities [21] and found that the transition state structure was stabilized by a hydrogen bond with one of the hydroxyl groups of β-cyclodextrin, which explained the rate acceleration. Castro et al. studied the effect of β-cyclodextrin on the hydrolysis of *N*-phenylphtalamide (Ph) and Nadamantylphtalamide (Ad) by the PM3 method [22]. Their result showed that all the β-cyclodextrin/Ad complexes are more stable than the β-cyclodextrin/Ph complexes. Recently Giuseppe Floresta et al. studied γ-cyclodextrin as a catalyst for the synthesis of 2-methyl-3,5diarylisoxazolidines with the PM3 method [23] and found that the transition states (TSs) coming from the nitrone in an E-configuration are about 3 kcal/mol more stable than that derived from the Z-one. In this paper, we performed computational work using both molecular mechanics, including free energy calculations and molecular dynamics (MD) simulations, and quantum mechanical (QM) calculations to investigate Diels-Alder reactions with cyclodextrins as catalysts. We studied the reaction of 9-anthracenemethanol (compound **1**) with *N*-cyclohexylmaleimide (compound **2a**) (Figure 2) catalyzed by β-cyclodextrins. The study describes a new mechanism and explains why methyl-β-cyclodextrin outperforms β-cyclodextrin to promote the reaction.

## 2. Results and Discussion

### 2.1. Free Energy Calculation for Cyclodextrin and Substrate Binding

To perform as a catalyst, the cyclodextrin must form a close contact with a reactant(s). The cavity size of β-CD or dimethyl-β-CD is only large enough to accommodate one reactant, so we first searched for the reactant and β-CD/dimethyl-β-CD complex conformations and examined which reactant bound to a cyclodextrin first by computing the binding free energies of **1** or **2a** to β-CD or dimethyl-β-CD with the VM2 method [24].

The calculated binding free energies and decompositions suggest that at the initial step of the catalyzed reaction, compound **2a** is more likely to bind to cyclodextrins than compound **1** (Table 1). The computed binding affinity of **1** to β-CD and dimethyl-β-CD was −2.71 and −4.09 kcal/mol—at least 2 kcal/mol weaker than **2a** binding to the catalysts. Compound **2a** had a similar computed binding affinity with both β-CD and dimethyl-β-CD, −6.58 and −6.17 kcal/mol, respectively. Experimental NMR proton chemical shifts suggested that the binding of **2a** with dimethyl-β-CD yielded a change in the chemical shift by 0.078 ppm, as compared with 0.047 ppm from **2a** binding with β-CD [15]. The NMR measurement did not explicitly quantify the binding affinity between **2a** and β-CDs, but the changes in the chemical shift suggested that **2a** prefers binding to dimethyl-β-CD. Although our free energy calculations yielded a similar binding free energy for **2a** binding to both CDs, calculations show stronger enthalpic attraction between **2a** and dimethyl-β-CD.

**Figure 3:**
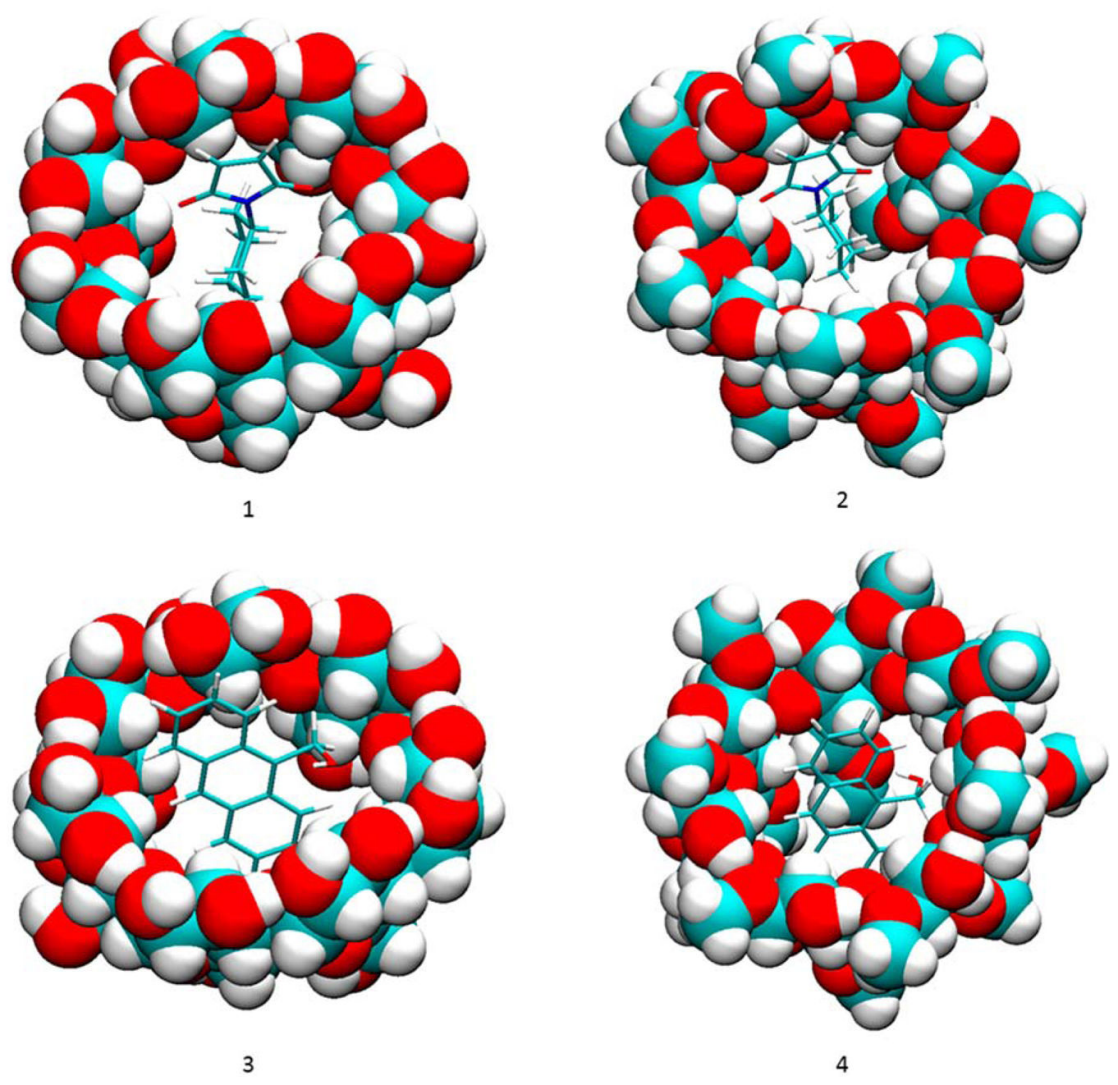
Binding modes of compounds **1** and **2a** to cyclodextrins by VM2. (**1**) **2a** and β-CD; (**2**) **2a** and dimethyl-β-CD; (**3**) **1** and β-CD; (**4**) **1** and dimethyl-β-CD. In all figures, cyclodextrins are represented as balls and compounds **1** and **2a** are represented as sticks. In both representations carbon is in cyan, oxygen in red and hydrogen in white.

The binding modes computed by VM2 showed no significant Hbonds between compound **2a** and the cyclodextrins, so Hbond formation may not be the sole functional structure of the catalyst. As shown in Figure 3, the cyclohexyl group of **2a** snugly sits right in the hydrophobic cavity of the cyclodextrin, and the maleimide moiety stays at the wide rim side (the side with secondary hydroxyl groups) of the cyclodextrin, whereas the anthracene rings are in the cavity and remain vertical to the macrocycles of the cyclodextrin. The principal axes of the reactants are aligned well with those of the cyclodextrin to make tight contacts with each other. The main driving force for the binding is van der Waals interactions, which is expected because of the non-polar cavity of the cyclodextrin. The value is similar for all complexes as well. Surprisingly, we observed no Hbonds between **2a** and the cyclodextrin in all conformations. In the complex with **2a**, the secondary hydroxyl groups on β-CD still kept a perfect intra-molecular hydrogen bonding network. The poor solubility of β-CD is believed to be attributed to this stable hydrogen bonding network and methylation is used to break this network to increase the solubility [25]. However, the hydroxyl groups on dimethyl-β-CD are not able to interact with the carbonyl group on **2a** either. Because of the lack of favorable hydrogen bonding interactions, the overall electrostatic contribution (ΔE_Coulomb_ + ΔE_PB_) is positive for the binding between the reactants and the cyclodextrin. In sharp contrast to the hypothesis that Hbonds between the carbonyl groups of **2a** and cyclodextrins play a key role in the catalysis reaction between compound **1** and maleimide derivatives including **2a** [15], our VM2 results do not support this hypothesis.

### 2.2. Conformational Fluctuations Modeled by MD Simulations

The VM2 method provides multiple local and global energy minima conformations, which also reveals that the systems are highly flexible. In solution, the systems rarely stay in their energy minima conformation, and each molecule constantly fluctuates. Therefore, we ran 300-ns all-atom unbiased MD simulations in an explicit solvent model for the complexes and found more diverse conformations.

We first examined the binding strengths and conformations of the complexes of **2a** and β-CD/dimethyl-β-CD, and the results confirmed a stronger enthalpic interaction between **2a** and dimethyl-β-CD than with β-CD due to more favorable van der Waals interactions. We calculated the interaction energies between the reactant and the cyclodextrins with the Molecular Mechanics Poisson-Boltzmann Surface Area (MMPB/SA) method to examine their intermolecular attractions (Table 2). The MMPB/SA energies are consistent with the VM2 results in that dimethyl-β-CD has a stronger enthalpy contribution to the binding with **2a** than β-CD. The dominant contributor is the van der Waals interaction, and the overall electrostatic interaction is positive. Table 3 compares the average representative distances between the geometric center of **2a** or its moieties and the center of a cyclodextrin. Larger positive distances indicate that a reactant is farther from the center of the cyclodextrin cavity and near the wide rim of a cyclodextrin. In contrast, a small or negative value indicates that the reactant **2a** binds deeply into the pocket of a cyclodextrin toward the narrow rim. In general, **2a** binds deeper into non-polar pocket of β-CD than that of dimethyl-β-CD sampled by both methods. The MD sampling provides reasonable binding modes for **2a**, and the cyclohexyl group of **2a** forms a close contact inside the non-polar pocket of the cyclodextrin. In contrast with **2a** binding to β-CD, the compound binds near the top of dimethyl-β-CD, and the shallow binding pose allows the five-membered maleimide ring to be exposed more to the solvent, thereby increasing the possibility for compound **1** to interact with **2a** and dimethyl-β-CD. This binding mode also brings the carbonyl group closer to the secondary hydroxyl groups of dimethyl-β-CD to possibly form an Hbond.

We examined the proportion of Hbonds between *N*-cyclohexylmaleimide and the hydroxyl groups of the cyclodextrins during the MD simulations and found very few. This observation does not support the hypothesis that these Hbonds play an important role in the catalysis as claimed by Chaudhuri et al. [15]. For the dimethyl-β-CD and **2a** complex, the proportion is negligible (0.67%) and for β-CD, the proportion is only slightly higher (4.10%). The very low percentage indicates that these Hbonds are not stable, which is consistent with our VM2 results, and we find little to no Hbonds in all energy minima. Our results suggest a different mechanism other than a substrate forming the Hbonds with cyclodextrins to promote the Diels-Alder reaction. Assuming that the Hbonds play an important role in enhanced catalysis, our results show that β-CD can have a slightly higher possibility of forming Hbonds, which should result in a better catalyst. However, experiments did not support this hypothesis either.

**Figure 4:**
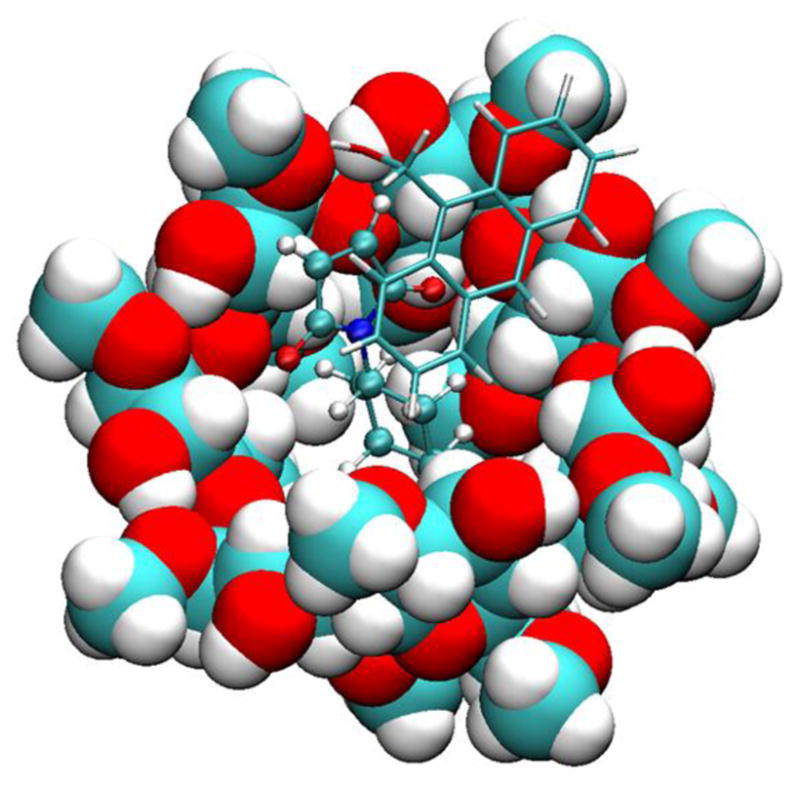
Binding mode of compound **1** with **2a** and dimethyl-β-CD. Compound **1** is rendered in licorice, **2a** in CPK, and dimethyl-β-CD in VDW.

**Figure 5:**
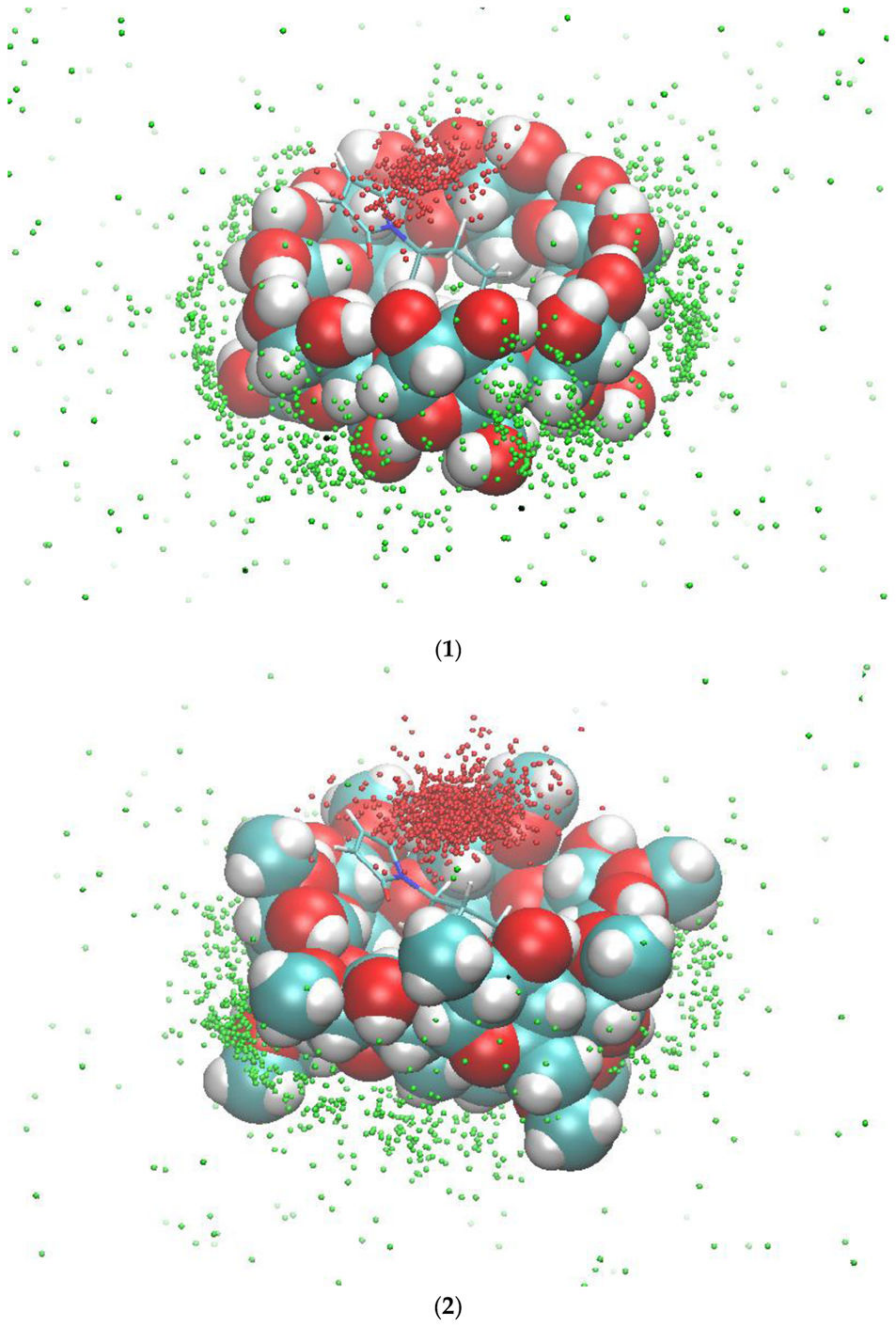
The occurrences of compound **1** inside the binding site of β-CD (**1**) and dimethyl-β-CD (**2**) in the presence of compound **2a**. The red dots (inside the binding site) and the green dots (outside the binding site) are the geometric centers of compound **1** during the 300-ns MD runs. The initial conformations of compound **2a** and β-CD/dimethyl-β-CD for the MD runs are used as a reference.

We then investigated the simultaneous binding of compounds **1** and **2a** to β-CD or dimethyl-β-CD. The MMPB/SA results show that the binding of **1** and **2a** with cyclodextrins has a more favorable energy than the binding of **2a** alone (Table 2); as such, after **2a** binds to the cavity of the cyclodextrin, **1** would also spontaneously bind to the complex. Therefore, cyclodextrins can assist in geometrically pre-organizing the two reactants for the Diels-Alder reaction, and the binding is driven by van der Waals interactions. Dimethyl-β-CD binds **1** and **2a** more tightly than does β-CD because it has more non-polar groups to contribute to the van der Waals energy. Figure 4 shows the typical orientation of **1** when interacting with **2a** and dimethyl-β-CD. One end of its anthracene ring and the maleimide ring of **2a** forms a π-π stacking interaction, and the other end is in contact with the methyl-oxyl group on dimethyl-β-CD. We further inspected the dynamics of compound **1** in the complexes. Binding of **1** to the complex of **2a** and the CDs is not as stable as binding to the CDs without **2a** because **1** frequently moved in and out of the binding site in the complex of **2a** and CDs. **2a** and **1** are more stable in dimethyl-β-CD than β-CD. The tri-molecule complex with dimethyl-β-CD was present at 34.9% of the MD simulation time, which is significantly longer than the tri-molecular complex with β-CD, which is only present at 8.1% of the MD time (Figure S2). In addition to the percentage, we examined the length of formation of the tri-molecular complex. The 1–2a–dimethyl-β-CD complex can last as long as 60 ns, in contrast with the 1–2a–β-CD complex which formed for less than 10 ns. Figure 5 records the locations of the geometric center of 1 with the initial conformation of **2a** and the cyclodextrin as a reference during 300 ns. Notably, the initial conformations are the bound states of both reactants and β-CD/dimethyl-β-CD, as described in Section 2.2. These results suggest that dimethyl-β-CD is able to keep both reactants in its binding site for much longer than β-CD.

### 2.3. Explanation of Enhanced Catalysis by QM Calculations

Our molecular mechanics calculations suggest that cyclodextrins help pre-organize the two reactants in the correct geometry arrangement. To gain a better understanding of whether such a pre-organization can actually lower the activation barrier and promote a Diels-Alder reaction, we used large-scale QM calculations for five reactions: the Diels-Alder reaction of **1** with **2a** without cyclodextrins, and **1** and **2** plus dimethyl-β-CD or β-CD with or without the Hbond formation. As an initial effort, a semi-empirical PM3 method was used to locate the TSs and the corresponding Intrinsic Reaction Coordinates (IRC) with and without β-CD or dimethyl-β-CD. Despite its limitations, the PM3 method correctly characterizes the bond breaking and formation of Diels-Alder reactions [26] and is a practical method for large systems as in this work. In addition, our QM calculations were also carried out at the ωB97XD/6-31G(d) level of theory to compute the optimized TS, reactant, and product geometries for all five reactions. We have specifically chosen to use the ωB97XD functional for this work since this QM method incorporates dispersion effects in conjunction with a large amount of exact exchange—both effects (particularly the latter) are required for accurately calculating reaction barriers in Diels-Alder reactions [27].

**Figure 6:**
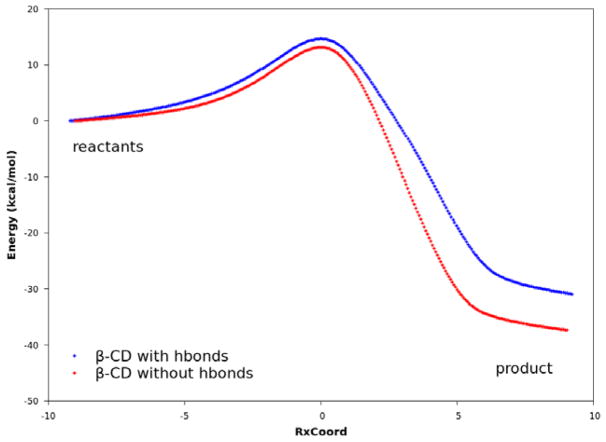

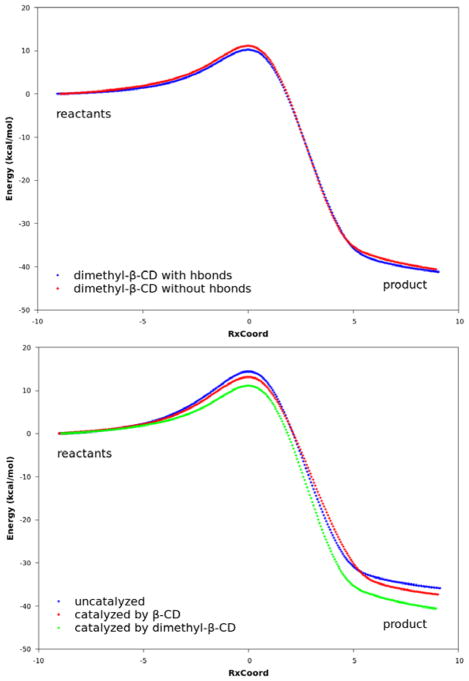
Reaction paths obtained with DFT. In all plots, the curves are normalized to set the reactant energies at zero. Top: reaction paths for the reaction catalyzed by β-CD, with and without hydrogen bonds. Middle: reaction paths for the reaction catalyzed by dimethyl-β-CD, with and without hydrogen bonds. Bottom: reaction paths for uncatalyzed reaction between compounds **1** and **2a**, reaction catalyzed by β-CD, and reaction catalyzed by dimethyl-β-CD. No hydrogen bonds exist between compound **2a** and the cyclodextrin.

**Figure 7:**
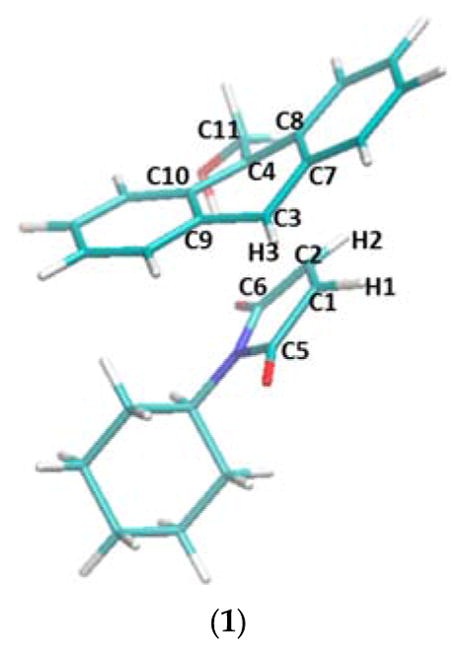

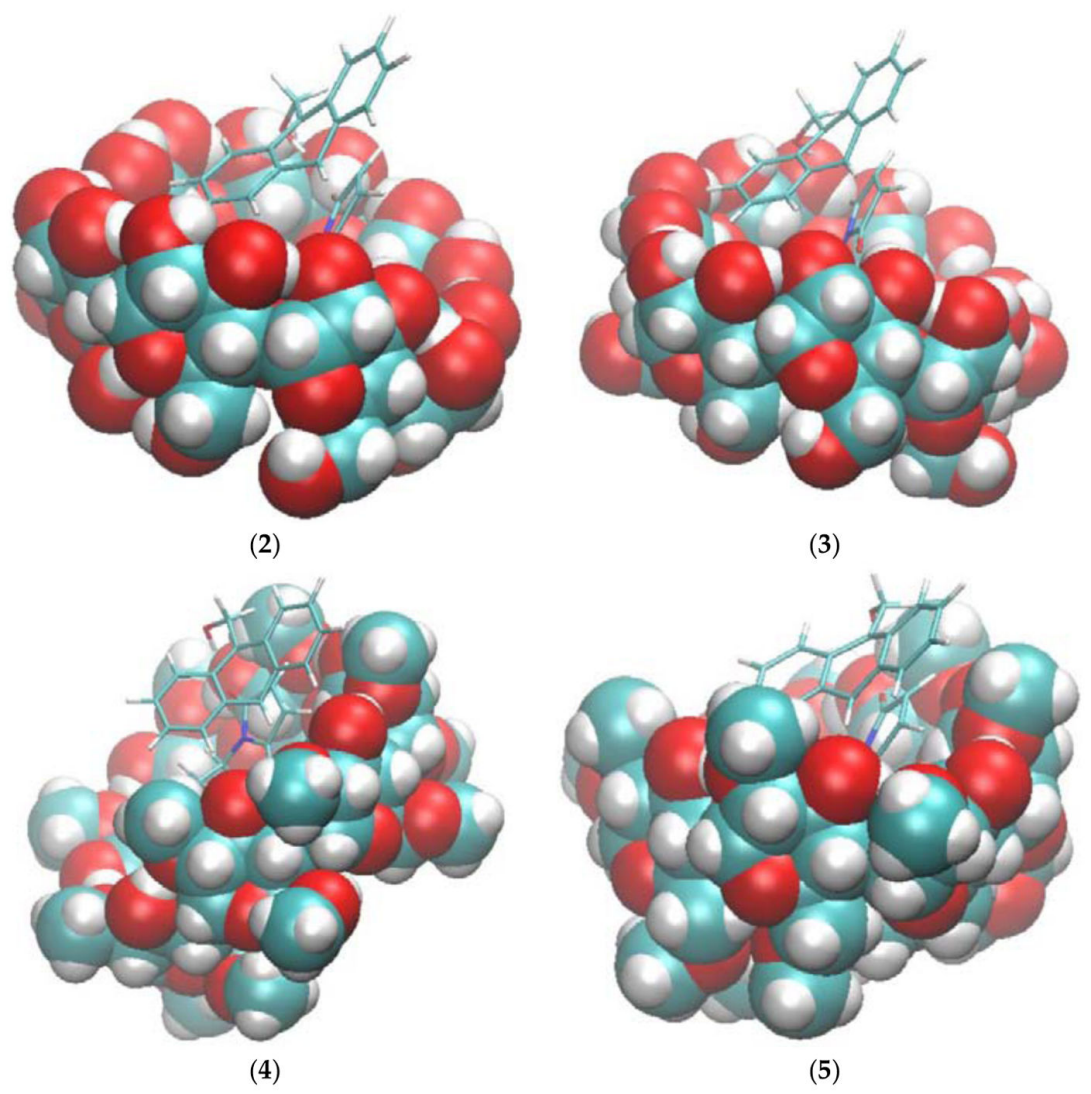
The transition states of five reactions optimized at the ωB97XD/6-31G(d) level of theory. (**1**) Transition state structure in the non-catalyzed reaction between compounds **1** and **2a**. Atom names used in Table 4 are labeled in this structure. (**2**) Transition state structure in the reaction catalyzed by β-CD, with hydrogen bonds between the two carbonyl groups of **2a** and β-CD. (**3**) Transition state structure in the reaction catalyzed by β-CD, without the hydrogen bonds. (**4**) Transition state structure in the reaction catalyzed by dimethyl-β-CD, with hydrogen bonds between the two carbonyl groups of **2a** and dimethyl-β-CD. (**5**) Transition state structure in the reaction catalyzed by dimethyl-β-CD, without the hydrogen bonds.

The QM calculations from both the PM3 method and the DFT method suggest that Hbonds play a nearly negligible role in lowering the activation energy (Figure 6 and Figure S3). Structures with the lowest energies during the MD runs were optimized with the geometry optimization method using an energy-represented direct inversion in the iterative subspace (GEDIIS) algorithm which was subsequently used for searching the TS geometries of the cyclodextrin-catalyzed reactions with both the PM3 method and density functional theory (DFT) at the ωB97XD/6-31G(d) level of theory. The optimized structures of the TSs and their key geometric parameters are depicted in Figure 7 and Table 4 (DFT), and Figure S4 and Table S1 (PM3), which show that the non-catalyzed TS configuration is largely maintained in the catalyzed reactions. In all of the TSs, the lengths of the two new “covalent bonds” between **1** and **2a** are similar, as are the improper dihedral angles at the four carbon atoms where these two new bonds are to be formed. Therefore, the catalytic effects of cyclodextrins are not from the direct participation of bond formation at TS. In addition, the IRCs with and without Hbonds with both catalysts (Figures 6 and S3, top and middle respectively) are similar. The reactions have slightly lower activation energies by no more than 2.0 kcal/mol when Hbonds exist between catalysts and **2a**. This finding suggests that hydrogen bonding does not play a significant role in catalyzing the reaction. Interestingly, when the IRC of the uncatalyzed reaction is plotted against that of the catalyzed reactions without Hbonds (Figure 6 and Figure S3 bottom), they look quite similar. For instance, the activation energies by the PM3 method are 28.20, 28.75, and 27.28 kcal/mol for reactions without a catalyst, with β-CD, and with dimethyl-β-CD, respectively. Because IRC calculations report only the potential energies for the reaction systems, the high similarity in potential energies suggests that the catalytic power of cyclodextrins may be related to the entropy.

As such, we calculated the activation free energy and its enthalpy and entropy components for all five reactions (Table 5 and Table S2 for DFT and PM3 methods, respectively). Surprisingly, the activation free energy for the non-catalytic reaction was 16.89 kcal/mol from our DFT calculations, which is lower than those of the catalyzed reactions. This result is not consistent with the experimental observation [15]. In contrast, the activation free energy for the non-catalytic reaction was 49.04 kcal/mol from the PM3 method, which is in the range of experimental activation free energies for typical Diels-Alder reactions [28,29]. Also, in Table S2, cyclodextrins lower the energy barriers significantly, and dimethyl-β-CD performs better than β-CD, in agreement with the experimental observation [15]. The calculated activation free energies can be decomposed into enthalpy and entropy terms. Most importantly, both the QM methods show that the entropy term −TΔS is the dominant factor in differentiating the activation energies. For the second sets, entropy drops from 16.03 kcal/mol in the non-catalyzed reaction to 2.98 kcal/mol in the β-CD catalyzed reaction, and to 0.5 kcal/mol in the dimethyl-β-CD catalyzed reaction according to the DFT results. From the PM3 calculations, the entropy values are 17.28, 5.00, and 1.04 kcal/mol for the above three reactions, respectively. The PM3 energies (Table S2) show that the first sets of both β-CD and dimethyl-β-CD complexes have a lower enthalpy term than the second set, so the Hbonds may help stabilize the transition state. However, the presence of Hbonds lowers the free energy by only less than 2 kcal/mol for both catalysts. As compared with the 10 to 20 kcal/mol stabilization of the activation energy in the catalyzed reactions, the impact of Hbonds is small. The energies from the DFT calculations (Table 5) show no difference with or without Hbonds in the β-CD catalyzed reaction. In the dimethyl-β-CD catalyzed reaction, the system with Hbonds has an enthalpy that is 10 kcal/mol lower than the one without Hbonds. However, the enthalpy computed from DFT for the non-catalyzed forward reaction may not be highly accurate because the non-catalytic reaction has an unreasonably low enthalpy of 0.86 kcal/mol, compared with all other sets of molecules. This low enthalpy is not consistent with the energies on the IRC curve either (Figure 6). As a comparison, the results from the PM3 method are self-consistent and in agreement with the experiments (Table S2). Regardless of the presence of Hbonds, the TSs in the dimethyl-β-CD catalyzed reaction have a lower enthalpy than those in the β-CD catalyzed reaction as obtained from both QM methods. Therefore, the binding of compounds **1** and **2a** to cyclodextrins acts as a pre-organization and remarkably reduces the entropy penalty to the activation free energy for the transition state of **1** and **2a**.

The above results show that instead of directly participating in chemical bonding at the transition state, the pre-organization provided by complexation between compounds **1** and **2a** and CDs lowers the activation energy of the Diels-Alder reaction. The catalytic effect is achieved by the reduction of ΔS when going from the reactant state to the reaction barrier. The limitations of this QM study also deserve a few comments. Because of the existence of multiple conformers of cyclodextrins, there may be multiple reaction pathways and TS structures. Because cyclodextrins do not participate in the chemical bonding at the barrier, its configurations are not expected to change significantly during the reaction. Therefore, the configuration effect is largely cancelled in the activation energy calculations. Furthermore, the initial configurations used in the QM calculations are those with the lowest energies from the MD runs, so they may represent the configuration with the largest statistical weight. Nevertheless, we could further investigate the configurational contributions of cyclodextrin to the activation barrier. Since the thermodynamic functions by both the QM methods do not take into account configurational effects over multiple conformations, the calculated entropies are also underestimated as compared to reality. Solvent effects were not considered in this QM study, and they are expected to be similar for both catalyzed and un-catalyzed reactions because the reaction coordinates of the TSs (i.e., eigenvectors) are similar for both catalyzed and un-catalyzed reactions. Omitting solvent effects should not affect the conclusions of this paper; however, our QM activation energies for the reaction barrier were in the range of the experiment and correctly ranked the CD catalysts. These observations suggest that the QM results are reasonable.

In summary, the major findings from this MM and QM study are as follows: (1) entropy plays a significant role in lowering the activation free energy barrier in the Diels-Alder reactions catalyzed by cyclodextrins; (2) hydrogen bonding, although lowering the barrier to some extent, is less important than the entropy contribution; and (3) dimethyl-β-CD is more efficient than β-CD in catalyzing the reaction.

Interestingly, our findings are echoed by a very recent paper [30]. Henrik Daver et al. studied the cycloaddition reaction between phenyl acetylene and phenyl azide inside a synthetic, self-assembled capsule with DFT, and concluded that the reduction of the entropic cost of bringing together the reactants are the major contributors to the rate acceleration compared to the background reaction.

## 3. Materials and Methods

**Figure 8:**
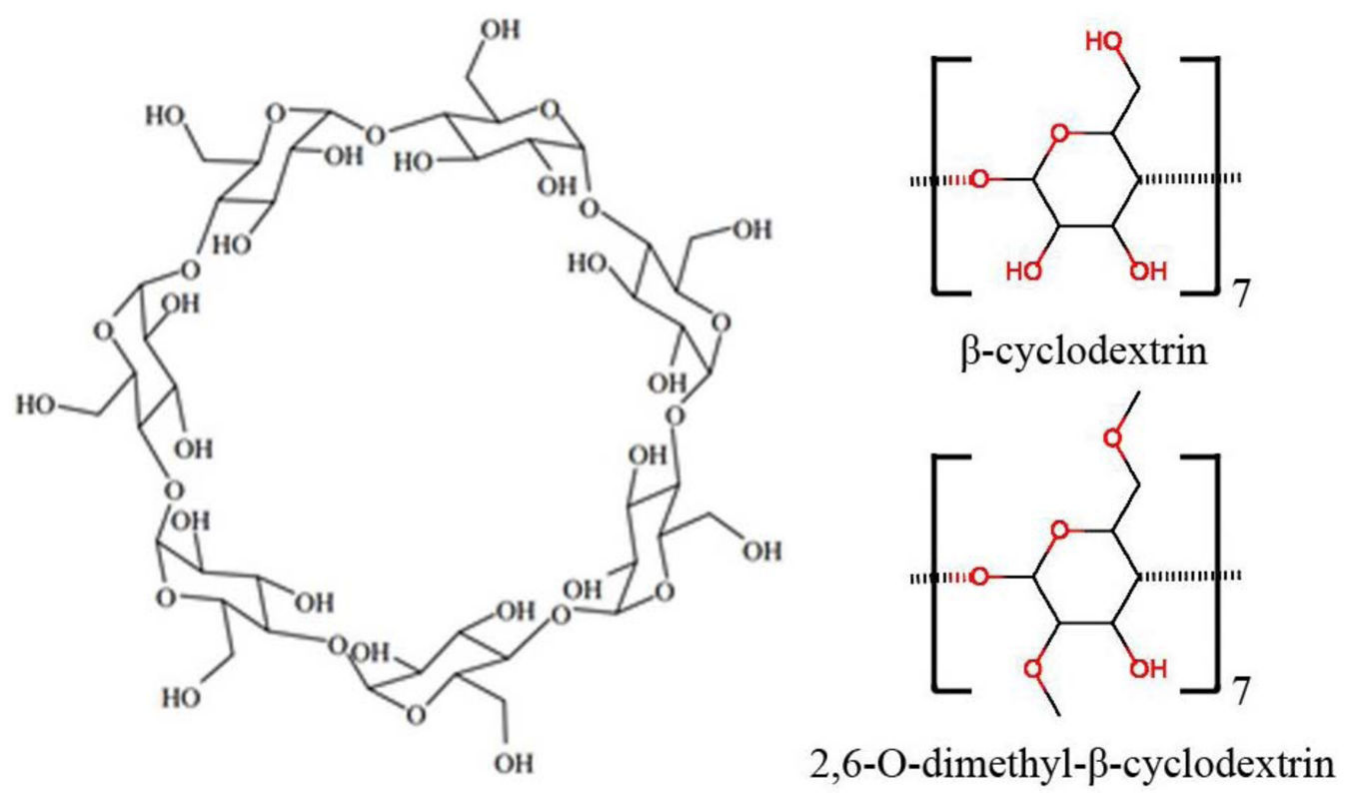
Molecular structure of β-cyclodextrin. The shape of the cyclodextrin macrocycle can be described as a truncated cone, with a narrow rim presenting primary hydroxyl groups and a wide rim presenting secondary hydroxyl groups on the glucose residues.

Each α-D-glucopyranoside on the cyclodextrin has one primary hydroxyl group and 2 secondary hydroxyl groups (Figure 8). All the commercially available methylated β-cyclodextrins are mixtures of various isomers and homologues except trimethyl β-cyclodextrin. Since the study by Chaudhuri et al., showed an average of ~1.8 methyl groups in methyl-β-cyclodextrin, and the primary hydroxyl group was more active than secondary hydroxyl groups in methylation, we assume that the major component of methyl-β-cyclodextrin is 2,6-O-dimethyl-β-cyclodextrin. For these reasons, we investigated the interactions of the two reactants (**1** and **2a**) in the Diels-Alder reaction with the catalysts β-cyclodextrin (β-CD) and 2,6-O-dimethyl-β-cyclodextrin (dimethyl-β-CD) by the computational methods briefly described below. For molecular mechanics calculations, the q4md force field [31] and General Amber Force Field (GAFF) [32] were applied to cyclodextrins and the two reactants, respectively. The figures of the molecule conformations in this paper were generated using Visual Molecular Dynamics (VMD) [33].

### 3.1. Free Energy Calculations with the VM2 Method

The initial conformations of the complexes for the VM2 calculations were obtained by using an in-house docking script. The VM2 method [24] provides binding affinities by computing the standard chemical potentials of the free receptor, the free ligand, and their complexes and taking the differences to obtain the standard free energy of binding. The standard chemical potential of each molecular species is obtained as a sum of the contributions from the low-energy conformations of the species. These conformations are identified with the Tork conformational search algorithm [34], and a symmetry-corrected method ensures that no conformation is double-counted in the free energy sums [35]. The contribution of each unique energy well to the free energy is computed with an augmented form of the harmonic approximation, the Harmonic Approximation/Mode Scanning (HA/MS) method [36]. The ligand, the receptor, and their complex are treated as fully flexible during these calculations.

### 3.2. Unbiased MD Simulations

We performed four sets of MD simulations for the 2a-β-CD complex, 2a-dimethyl-β-CD complex, and pre-organized **1**–**2a** reactant complexes binding to β-CD and dimethyl-β-CD, respectively. The initial conformations of the complexes of cyclodextrins and compound **2a** for the MD simulations were the lowest energy conformations obtained from VM2. For the complexes of cyclodextrins with both reactants, the initial conformations were obtained by the following method: the reaction product was added in place of **2a** in the initial conformations of the complexes of the cyclodextrins and **2a**, then the two single bonds connecting **1** and **2a** in the product were removed and the whole system was energy minimized.

The Amber 14 package with GPU implementation [37,38] was used for the MD simulations for the complexes. Minimization on the hydrogen atoms and the entire complex was applied for 500 and 5000 steps, respectively. After being solvated with a rectangular TIP3P water box [39], the edge of the box was at least 12 Å away from the solutes. The system went through a 1000-step water and 5000-step system minimization to correct any inconsistencies. Next, we relaxed the system by slowly heating it during an equilibrium course of 10 ps at 200, 250, and 298 K. We performed a production run in an isothermic-isobaric (NPT) ensemble with a 2-fs time step. The Langevin thermostat [40,41], with a damping constant of 2 ps-1, was used to maintain a temperature of 298 K. The long-range electrostatic interactions were computed by the particle mesh Ewald method [42] beyond a 8 Å distance. We collected the resulting trajectories every 2 ps. Finally, the SHAKE algorithm [43] was used to constrain the hydrogen atoms of water during the MD simulations. We performed 300 ns of MD production runs on each complex by using CPU parallel processing and local GPU machines. Finally, the trajectories were collected and analyzed at intervals of 20 ps.

In this work, an Hbond (X-H … Y) was considered formed if the distance between H and Y was smaller than 2.2 Å and the complimentary angle of X-H … Y was smaller than 90° (Figure S1). We used an in-house script to post-process the trajectories for direct Hbonds between **2a** and cyclodextrins. The occurrence percentage of an Hbond was calculated as the number of the frames containing the Hbond divided by the total 3000 frames.

We computed the RMSD of compound **1** with respect to the initial conformation in the MD runs to determine whether compound **1** bound to the cavity of a β-CD or not. The RMSD data for compound **1** formed several clusters in this figure. The one with RMSD < 5 Å corresponded to the binding of **1** with **2a** and β-CD/dimethyl-β-CD.

### 3.3. Quantum Mechanics Calculations with the PM3 Method

Reaction paths and transition states (TSs) with and without β-CD and dimethyl-β-CD catalysts were calculated with both the PM3 method and the DFT method at the ωB97XD/6-31G(d) level of theory in the G09 package [44,45]. Aqueous solvent was not included in the present calculations. The Gibbs free energies as well as the enthalpy and entropy components of reactants and TS were calculated with standard statistical thermodynamics equations [46] at 298K using the optimized molecular structures. For example, entropy was calculated with Equation (1), where the partition function, *q*, is obtained by including contributions from translational, rotational, vibrational, and electronic degrees of freedom.

(1) $$S={Nk}_{B}+{Nk}_{B}ln\;\left(\frac{q(V,T)}{N}\right)+{Nk}_{B}T\;{\left(\frac{\partial \mathrm{lnq}}{\partial T}\right)}_{V}$$

The activation free energies were obtained by computing the difference between the free energies of the reactants and the TS. Molecular structures were optimized by GEDIIS method [47] in G09.

We performed two sets of QM calculations for compounds **1** and **2a** with β-CD or dimethyl-β-CD complexes with two β-CD conformations. The first set involved a conformation with Hbonds between the two carbonyl groups of **2a** and the catalyst, and the other set did not have Hbonds between **2a** and β-CD or dimethyl-β-CD. We also performed calculations for compounds **1** and **2a** without β-CDs. Complex structures with the lowest energies in the MD runs were selected as initial structures for searching the TS of a cyclodextrin-catalyzed reaction and geometry optimization. The Berny algorithm [48,49] was used for locating the TSs. After the TSs were identified, the energy profile along the reaction coordinate connecting reactants, TS, and products was calculated using the IRC. We used a step size of 0.02 amu^1/2^-Bohr and 150 steps were run in both forward and reverse directions along the reaction path.

## 4. Conclusions

Dimethyl-β-CD could catalyze Diels-Alder reactions of 9-anthracenemethanol with a variety of *N*-substituted maleimides under mild reaction conditions. To understand the mechanism, we performed MM and QM calculations. The driving force of the catalysis is not the Hbonds between cyclodextrins and **2a** because of the low occurrence of the Hbonds suggested by VM2 and MD results; furthermore, IRC calculations yielded small differences with or without the Hbonds. However, cyclodextrins, especially dimethyl-β-CD, do show a strong binding affinity with a pre-organized **1**–**2a** reactant complex and increase the probability of the Diels-Alder reaction. Moreover, QM calculations showed that cyclodextrins remarkably reduced the activation entropy. These findings depict a possible two-step catalysis mechanism by cyclodextrins: the first step is the complex formation between cyclodextrins and reactants, and the second step is the reaction of reactants inside the cavity of cyclodextrins to form the product. The formation of the complex is the key to the catalysis because it not only increases the rate that reactant molecules would meet each other but also lowers the entropy and thus the activation free energy of this reaction. Dimethyl-β-CD outperforms β-CD because of stronger van der Waals interactions with the pre-organized reactants and better performance in reducing the activation energy. Our QM results are in qualitative agreement with experiments on activation free energies of the Diels-Alder reactions and the relative catalytic activities for the systems under study.

## Supplementary Material

SI

## Figures and Tables

**Table 1:** Calculated free energies and energy decomposition analysis by the VM2 method for the complexes of reactants and cyclodextrins. Units are in kcal/mol. CD: cyclodextrin.

Complex	ΔG_calc_	ΔE_Valence_	ΔE_Coulomb_	ΔE_PB_	ΔE_NP_	ΔE_VDW_	ΔH	−TΔS
β-CD and **1**	−2.71	1.96	−8.04	15.69	−2.86	−25.27	−18.52	15.81
dimethyl-β-CD and **1**	−4.09	3.01	−8.50	14.84	−2.91	−26.06	−19.62	15.53
β-CD and **2a**	−6.58	0.39	−5.98	13.23	−2.76	−25.12	−20.23	13.65
dimethyl-β-CD and **2a**	−6.17	2.57	−11.25	15.28	−2.76	−25.22	−21.39	15.21

**Table 2:** MMPB/SA energies and energy decomposition analysis for the complexes of reactants with the cyclodextrins. Units are in kcal/mol. MMPB/SA: The molecular mechanics energies combined with the Poisson–Boltzmann and surface area continuum solvation method.

Complex	ΔE_Coulomb_	ΔE_PB_	ΔE_NP_	ΔE_VDW_	ΔH_MMPB/SA_
β-CD with **2a**	−7.24	17.23	−2.19	−23.07	−15.27
dimethyl-β-CD with **2a**	−3.38	13.35	−2.18	−24.51	−16.72
β-CD with **1** and **2a**	−16.00	27.79	−2.66	−25.88	−16.75
dimethyl-β-CD with **1** and **2a**	−5.51	19.17	−2.96	−31.09	−20.39

**Table 3:** Binding modes and conformation fluctuations sampled by MD and VM2. Average distances between geometric centers of compound **2a** or its moieties and the geometric center of the cyclodextrins were obtained from 300-ns MD runs and the VM2 results. Units are in Å. In the illustration above the table, the blue trapezoid represents the cyclodextrins; the black dot represents the center of the cyclodextrin, and the red dot represents the center of **2a** or its moieties; d1 is the distance between the centers of **2a** and the cyclodextrin; d2 is the distance between the centers of the cyclohexyl group and the cyclodextrin; d3 is the distance between the centers of the imide moiety and the cyclodextrin. The larger positive distances indicate that the ligand is farther from the center of the cyclodextrin cavity.

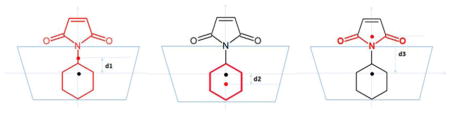			
Catalyst		MD	VM2
β-CD	d1	1.44 ± 0.70	0.38 ± 0.11
	d2	−0.03 ± 0.87	−1.39 ± 0.15
	d3	3.35 ± 0.68	2.14 ± 0.11

dimethyl-β-CD	d1	2.05 ± 0.73	1.42 ± 0.13
	d2	0.67 ± 0.87	−0.10 ± 0.17
	d3	3.95 ± 0.70	3.37 ± 0.12

**Table 4:** Key geometric parameters (bond lengths and improper dihedral angles) of the transition states optimized with DFT. Atom labels are depicted in Figure 7. Bond lengths are in Å and improper dihedral angles are in degrees.

No.	Bond C1–C3	Bond C2–C4	Improper C1–C2–H1–C5	Improper C2–H2–C1–C6	Improper C3–H3–C7–C9	Improper C4–C8–C11–C10
1	2.203	2.391	21.498	18.172	16.042	11.803
2	2.112	2.603	25.175	13.678	18.587	9.557
3	2.171	2.473	22.346	17.424	16.720	11.938
4	2.230	2.398	20.148	18.362	14.907	11.434
5	2.264	2.342	19.919	21.709	13.786	11.243

**Table 5:** Activation free energies and its enthalpy and entropy components obtained by DFT for the Diels-Alder reaction with and without cyclodextrin catalysts. Units are in kcal/mol.

Reaction	ΔG	ΔH	−TΔS
non-catalyzed forward reaction	16.89	0.86	16.03
non-catalyzed reverse reaction	48.06	48.66	−0.60
forward reaction catalyzed by β-CD, set 1 a	22.75	21.29	1.47
reverse reaction catalyzed by β-CD, set 1 a	47.83	49.44	−1.61
forward reaction catalyzed by β-CD, set 2 b	24.12	21.14	2.98
reverse reaction catalyzed by β-CD, set 2 b	48.67	49.92	−1.25
forward reaction catalyzed by dimethyl-β-CD, set 1 a	13.73	10.05	3.68
reverse reaction catalyzed by dimethyl-β-CD, set 1 a	48.51	51.50	−2.99
forward reaction catalyzed by dimethyl-β-CD, set 2 b	19.68	20.18	−0.50
reverse reaction catalyzed by dimethyl-β-CD, set 2 b	49.48	51.25	−1.77

a Transition state structure has hydrogen bonds between the two carbonyl groups of **2a** and the cyclodextrin;

b Transition state structure does not have hydrogen bonds.
