# Supplementary material for: An MM and QM Study of Biomimetic Catalysis of Diels-Alder Reactions Using Cyclodextrins
Source: Catalysts. Author manuscript; Available in PMC 2018 Jun 21. (PMC6011829; doi:10.3390/catal8020051)
Supplement: SI [file NIHMS959216-supplement-SI.pdf]

catalysts

MDPI

Supplemental materials: An MM and QM Study of Biomimetic Catalysis of Diels-Alder Reactions Using Cyclodextrins

Table S1. Key geometric parameters (bond lengths and improper dihedral angles) of the transition states optimized with the PM3 method. Atom names are in Figure 6 of the main text. Units are in Å for bond lengths and degrees for improper dihedral angles.

No.	bond C1–C3	bond C2–C4	improper C1–C2–H1–C5	improper C2–H2–C1–C6	improper C3–H3–C7–C9	improper C4–C8–C11–C10
1	2.130	2.298	23.119	20.047	16.033	13.738
2	2.125	2.316	22.883	19.641	15.879	13.229
3	2.127	2.312	23.413	19.531	16.184	13.394
4	2.141	2.297	22.869	21.064	15.451	13.417
5	2.140	2.283	22.885	20.346	15.911	13.947

Table S2. Activation free energies and its enthalpy and entropy components obtained by the PM3 method for the Diels-Alder reaction with and without cyclodextrin catalysts. Units are in kcal/mol.

Reaction	ΔG	ΔH	-TΔS
non-catalyzed forward reaction	49.04	31.76	17.28
forward reaction catalyzed by β-CD, set 1[a]	36.47	30.96	5.50
forward reaction catalyzed by β-CD, set 2[b]	38.32	33.32	5.00
forward reaction catalyzed by dimethyl-β-CD, set 1[a]	29.46	27.90	1.56
forward reaction catalyzed by dimethyl-β-CD , set 2[b]	29.17	28.13	1.04

a. Transition state structure has hydrogen bonds between the two carbonyl groups of 2a and cyclodextrin.

b. Transition state structure does not have hydrogen bonds.

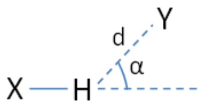

.

Figure S1. Definition of hydrogen bonds. X and Y are the donor and acceptor, respectively. d is the distance between the acceptor Y and the hydrogen, and *α* is the complimentary angle of X–H···Y. A hydrogen bond is formed if d is < 2.2 Å and *α* is < 90º.

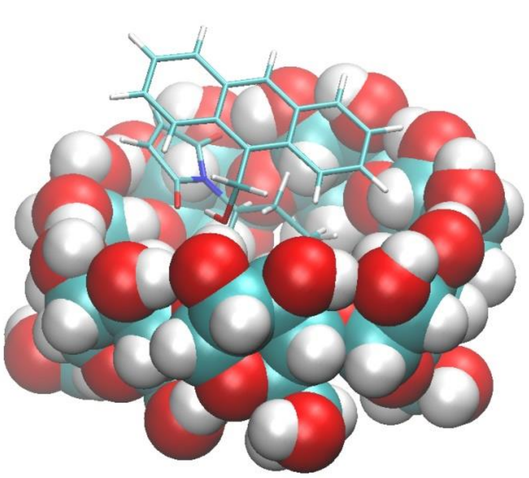

(**1**)

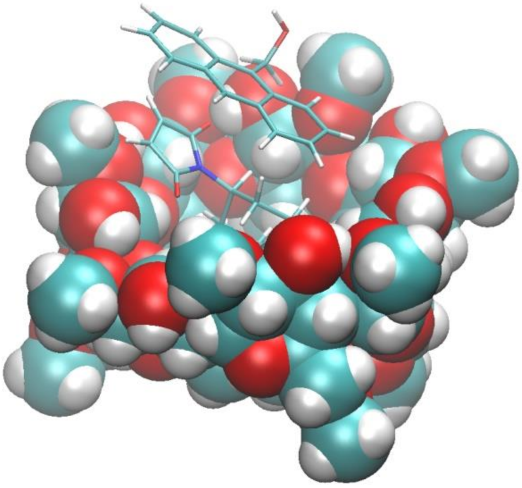

(**2**)

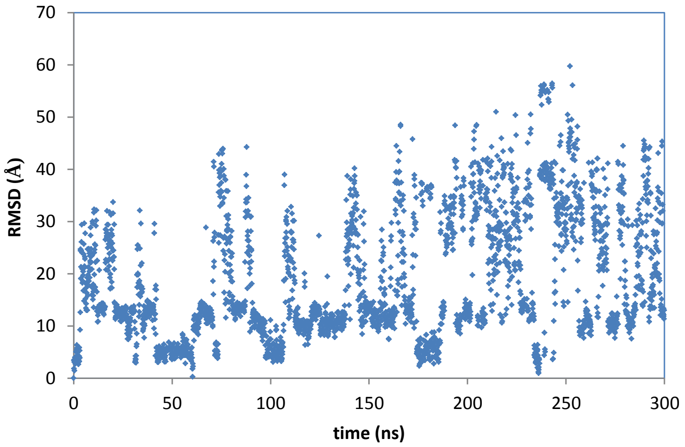

(**3**)

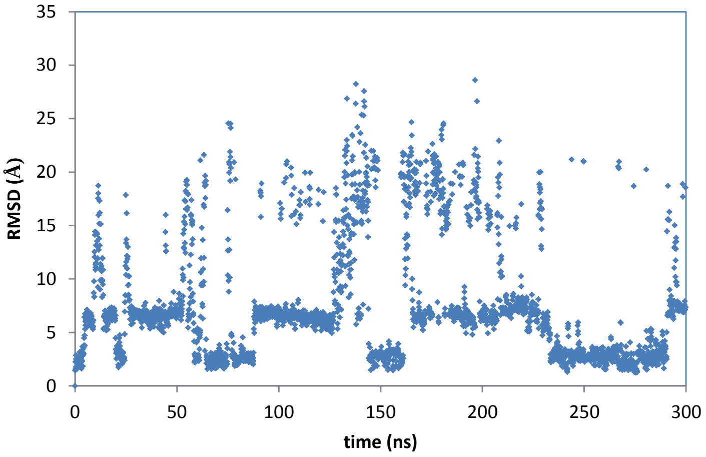

(**4**)

Figure S2. RMSD (**3**–**4**) of compound 1 with respect to the initial conformations (**1**–**2**) in the 300-ns MD runs. (**1**) (**3**): complex of β-CD with 1 and 2a; (**2**) (**4**): complex of dimethyl-β-CD with 1 and 2a.

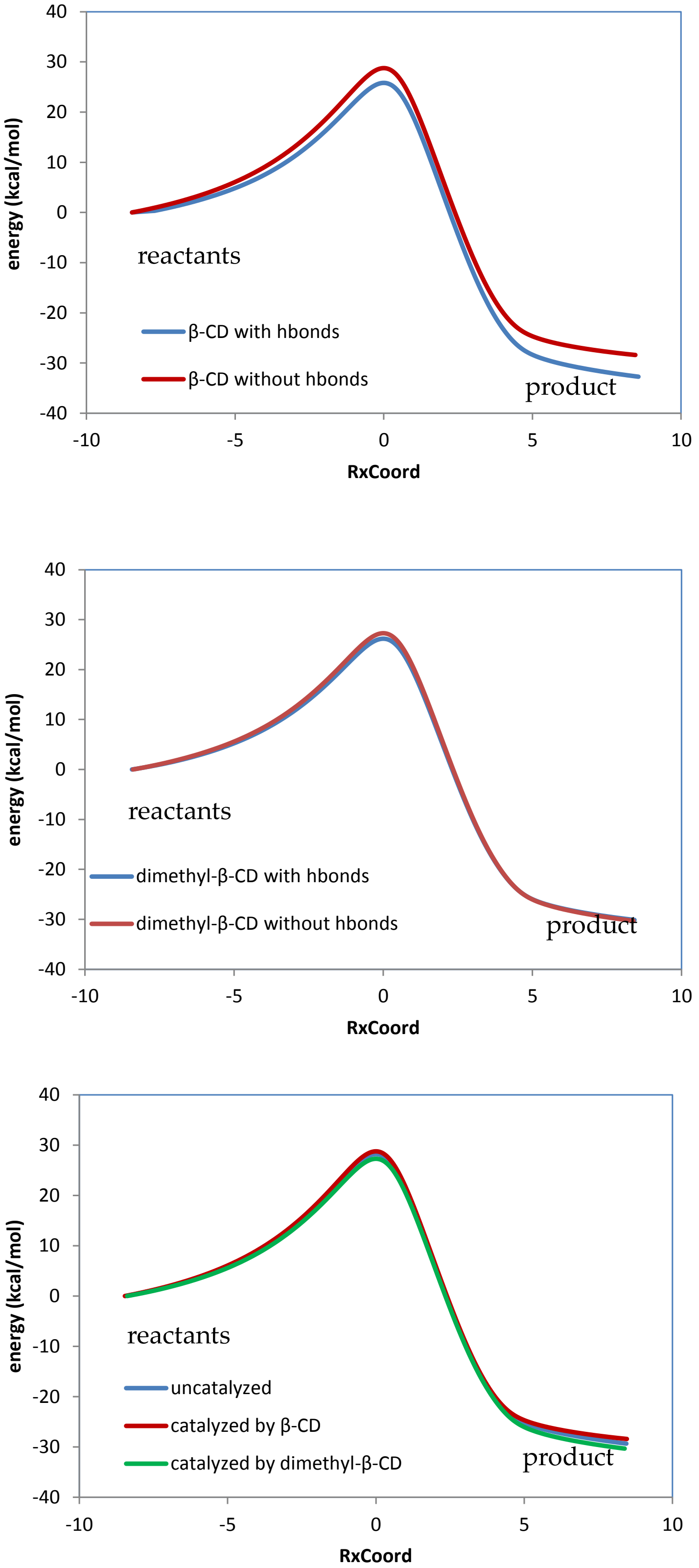

Figure S3. Reaction paths obtained with the PM3 method. In all plots, the curves are normalized to set the reactant energies at the zero of energy. Top: reaction paths for the reaction catalyzed by β-CD, with and without hydrogen bonds. Middle: reaction paths for the reaction catalyzed by dimethyl-β-CD, with and without hydrogen bonds. Bottom: reaction paths for uncatalyzed reaction between compounds 1 and 2a, reaction catalyzed by β-CD, and reaction catalyzed by dimethyl-β-CD. No hydrogen bonds exist between compound 2a and the cyclodextrin.

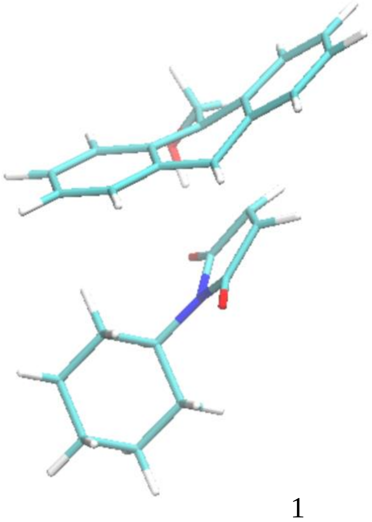

1

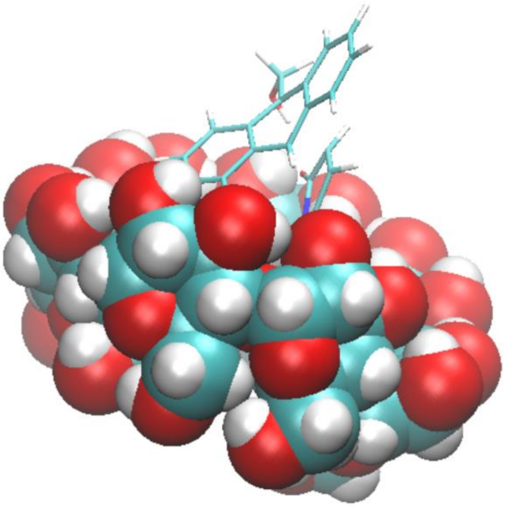

2

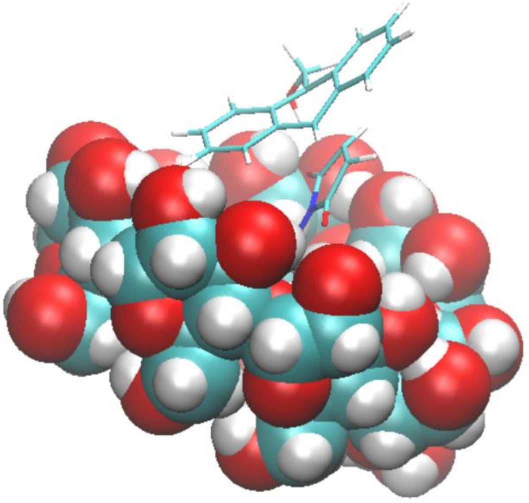

3

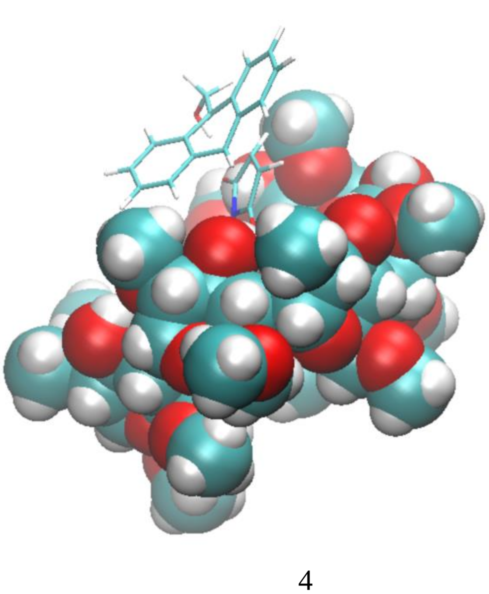

4

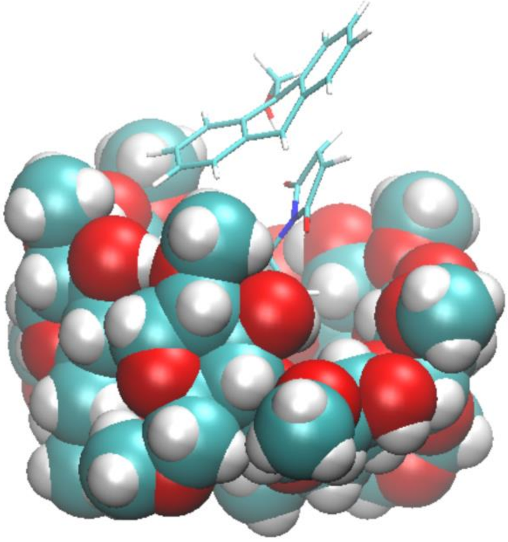

5

Figure S4. Transition states of five reactions optimized with the PM3 method. (**1**) Transition state structure in the non-catalyzed reaction between compounds 1 and 2a. (**2**) Transition state structure in the reaction catalyzed by β-CD, with hydrogen bonds between the two carbonyl groups of 2a and β-

CD. (**3**) Transition state structure in the reaction catalyzed by β-CD, without hydrogen bonds. (**4**) Transition state structure in the reaction catalyzed by dimethyl-β-CD, with hydrogen bonds between the two carbonyl groups of 2a and dimethyl-β-CD. (**5**) Transition state structure in the reaction catalyzed by dimethyl-β-CD, without hydrogen bonds.

© 2017 by the authors. Submitted for possible open access publication under the terms and conditions of the Creative Commons Attribution (CC BY) license (http://creativecommons.org/licenses/by/4.0/).
